# Use of a mobile health application by adult non-congenital cardiac surgery patients: A feasibility study

**DOI:** 10.1371/journal.pdig.0000055

**Published:** 2022-06-29

**Authors:** Sandra M. Ragheb, Anna Chudyk, David Kent, Mudra G. Dave, Brett Hiebert, Annette S. H. Schultz, Todd A. Duhamel, Rakesh C. Arora

**Affiliations:** 1 Max Rady College of Medicine, University of Manitoba, Winnipeg, Manitoba, Canada; 2 Department of Family Medicine, Rady Faculty of Health Sciences, University of Manitoba, Winnipeg, Manitoba, Canada; 3 St. Boniface Research Centre, Health Services & Structural Determinants of Health Research, Winnipeg, Manitoba, Canada; 4 Department of Cardiac Sciences, St. Boniface Hospital, Winnipeg, Manitoba, Canada; 5 Institute of Cardiovascular Sciences, St. Boniface General Hospital Albrechtsen Research Centre, Winnipeg, Manitoba, Canada; 6 Faculty of Kinesiology and Recreation Management, University of Manitoba, Winnipeg, Manitoba, Canada; 7 College of Nursing, Rady Faculty of Health Sciences, University of Manitoba, Winnipeg, Manitoba, Canada; Vanderbilt University, UNITED STATES

## Abstract

Mobile Health (mHealth) technologies are becoming integral to our healthcare system. This study evaluated the feasibility (compliance, usability and user satisfaction) of a mHealth application (app) for delivering Enhanced Recovery Protocols (ERPs) information to Cardiac Surgery (CS) patients peri-operatively. This single centre, prospective cohort study involved patients undergoing CS. Patients received a mHealth app developed for the study at consent and for 6–8 weeks post-surgery. Patients completed system usability, patient satisfaction and quality of life surveys pre- and post-surgery. A total of 65 patients participated in the study (mean age of 64 years). The app achieved an overall utilization rate of 75% (68% vs 81% for <65 and ≥65 years respectively). Pre-surgery, the majority of patients found the app easy to use (94%), user-friendly (89%), and felt confident using the app (92%). The majority also found the app’s educational information useful (90%) and easy to find (88%). 75% of patients reported that they would like to use the app frequently. This percentage decreased to 57% in the post-discharge survey. A lower percentage of patients ≥65 years indicated their preference for the app over printed information (51% vs 87%) and their recommendation for the app (84% vs 100% for >65 and <65 years respectively) in the post-surgery survey. MHealth technology is feasible for peri-operative CS patient education, including older adult patients. The majority of patients were satisfied with the app and would recommend using it over the use of printed materials.

## Author summary

Recently, mobile Health (mHealth) technologies have been utilized in healthcare as a novel medium to the delivery of patient knowledge sharing in the peri-operative phases of care. Given the old age of most cardiac surgery patient, several concerns have been raised regarding the usability of mHealth in this patient population due to technical difficulties or lack of interest in technology uptake. Thus, we sought to assess the feasibility of an mHealth to deliver patient education to this group. We found that overall cardiac surgery patients had a good uptake of the new technology, with only 5% attrition rate due to technical difficulties. The majority of enrolled participants reported high satisfaction with the app and rated the app highly in terms of its usability. The content of the app was also developed by the CS team based on evidence-based ERPs that were customized to the local clinical setting. Participant’s confidence in using the app without assistance contributed to the high adherence rate with app use. Our study demonstrates the feasibility of mHealth technology for peri-operative patient education in CS patient population.

## Introduction

Patient education has been part of healthcare for decades, and the content of educational materials along with mechanisms to impart information have evolved based on current evidence and new technologies. Currently, enhanced recovery protocols (ERPs) facilitate a standardized approach to peri-operative care encompassing evidence-based, multimodal, multidisciplinary interventions [1] that have been associated with improved patient outcomes [2]. Preoperatively, ERPs include counseling regarding smoking cessation, decreasing alcohol intake, nutritional screening and support, and optimization of chromic disease management. Post-operatively, early mobilization, early intake of fluids and solids, and multimodal approach to nausea, vomiting and pain control are part of ERPs [1]. Patient adherence to ERPs, however, is an important element to the success of such management strategies, given that various interventions often rely heavily on patient collaboration such as physical activity and nutritional intake peri-operatively [3,4]. This is particularly important for cardiac surgery (CS) patients who are more commonly older adults (≥ 65 years of age) with a level of functional decline and increased risk for morbidity and mortality post-surgery [5,6]. Effective patient education regarding their surgery and what to expect after surgery is crucial to increase patient-caregiver adherence to shared care plans [7] and improve patient outcomes, including hospital readmission rates [8,9] and quality of life [10]. Patients have similarly expressed the need for more comprehensive education regarding cardiac surgery ERPs to allow them to become active participants in their peri-operative care [11].

Effective patient education depends on several factors, including the medium, format, and amount of information delivered to patients. Individualized educational content in combined mode of delivery has been shown to enhance patient knowledge and behaviour change [12]. However, patients face various challenges with learning new information [13], with greater amounts of information, age-related memory changes and anxiety hindering recall of medical information [14]. Additionally, previous patient/caregiver focus groups conducted by our research team have indicated that patients are often overwhelmed by the amount of information they receive from their CS team (personal communication by Mackenzie King, 2017). Thus, there is a strong need to find novel ways to impart knowledge to patients.

Increasingly, mobile health (mHealth) technology has become integral to the functioning of our current healthcare system, specifically in the realm of patient education [12,13]. A systematic review by Mobasheri et al. showcased the utility of various smartphone applications (a.k.a. “apps”) in the peri-operative context [15]. A previous study demonstrated the effectiveness of such modality for CS patients [16]. The intervention considered in this study was limited to addressing pain management and mobility issues during patient hospitalization postoperatively [16]. In Canada, with 60% of adults over the age of 65 owning a smartphone [17], we have sought to adapt a mobile health (mHealth) platform or an “app” to enhance the delivery of patient and caregiver knowledge sharing in the pre- and postoperative phases of their care.

The use of mHealth apps for the purpose of cardiac surgery ERP patient education is a relatively new model of healthcare delivery [18]. Thus, the development of such technology for patient care requires various aspects to be considered. A user-centered design of mHealth has been proven to support its usability and patient engagement. This approach employs focus group sessions with intended end-users to identify their preferences for content and functions on the app [19]. Additionally, an app should directly address the needs of end-users to enhance its perceived usefulness by users. In the realm of surgical patients, information sharing regarding the peri-operative period (admission process instructions, surgery details, medication guidance, preoperative examination instructions, surgery complications, discharge instructions, and pictures/videos explaining the surgery) was strongly needed by patients to be included in the app [20]. Other functions that surgical patients indicated include appointment reminders, viewing test results, and communication with healthcare providers through the app [19,20].

This study aims to evaluate the feasibility (i.e., usability, adherence, and user satisfaction) of an mHealth app for delivering ERP information to patients undergoing CS and the impact of an mHealth app on clinical and patient-centered outcomes, including hospital readmission and quality of life.

## Materials and methods

### Study design

This single centre, prospective cohort feasibility study involved patients undergoing CS at St. Boniface Hospital, a tertiary care hospital in Manitoba, Canada. The study was approved by the University of Manitoba Research Ethics Board (H2018:458) and the St. Boniface Hospital Research Review Committee Research Ethics Board (RRC/2018/1813). Patients ≥ 18 years with a minimum of two-week wait time before their procedure (coronary artery bypass graft (CABG), valve, or combined CABG/valve surgeries only) were recruited between July 2019 and March 2020. Criteria for exclusion included known major cognitive impairment, inability to understand or read English, and lack of access to a smartphone/tablet or internet service. Patients were approached in the Cardiac Surgery Clinics and the in-patient cardiac care units to assess their eligibility for the study and obtain written informed consent. Once recruited, participants received the app at baseline (i.e., prior to surgery) and retained it until 6–8 weeks postoperatively.

A research assistant provided the participants (and the caregiver if present) with a brief educational session on proper app usage and technical support. A follow-up phone call one week after consent was provided to answer participant queries about the app. Participants completed patient satisfaction, app usability [21], and health-related quality of life (HRQoL) [22] surveys at four time points: 1) baseline (at the time of study consent), 2) pre-operative appointment, 3) pre-discharge, and 4) 6–8 weeks post-discharge during their clinic appointments or by phone (Appendix A in S1 Text). At all four time points, participants completed the health-related quality of life (HRQoL). Additionally, at the pre-operative and 6–8 week post-discharge appointments, participants completed the System Usabiliy and Patient Satisfaction surveys.

### mHealth app development

#### Patient engagement panel

**Fig 1:**
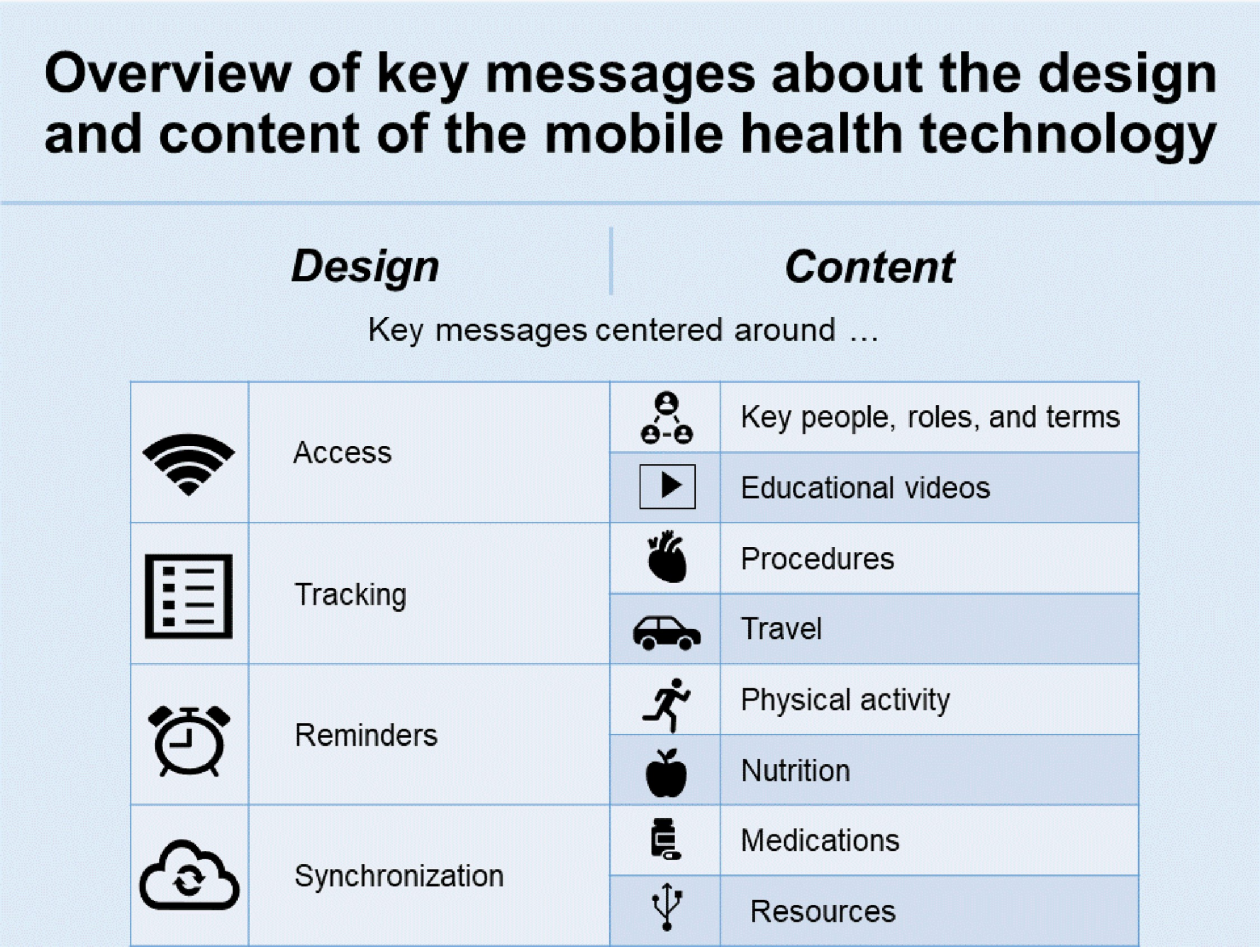
Patient Engagement Panel Key Messages.

Prior to the development of the information to be shared via the mHealth app, several patient engagement panel sessions were held. A patient engagement panel is a form of patient engagement in research [23] that seeks to include a group of patients as co-researchers by soliciting their input on the design, execution and/or application of study results. The engagement of patients in the development of mHealth technologies, particularly the design phase, can enhance the uptake of mHealth technologies by patients [24]. The patient engagement panel for this initiative consisted of 10 individuals (6 patients and four caregivers) who had previously undergone a CS procedure at our institution (2017 or 2018). The panel members had three in-person meetings, scheduled about two weeks apart. Group discussions were carried out to solicit experience-based input regarding the design and content of the app. Key messages about design and content of the mHealth app derived from the panel are found in Fig 1 [25]. Patients’ recommendations were relayed to the developer to incorporate into the app as supported by the platform’s current functionality.

### mHealth intervention

The aim of this app development was patient education and information sharing regarding the perioperative care and what to expect in order to increase adherence to shared care plans. Providing instructions on how to perform a specific behaviour is the most common behaviour change technique identified in previously studied mHealth Apps. Additionally, the app also included goal setting in the form of daily tasks to review certain tasks/topics at certain timelines of their surgical journey [26].

**Fig 2:**
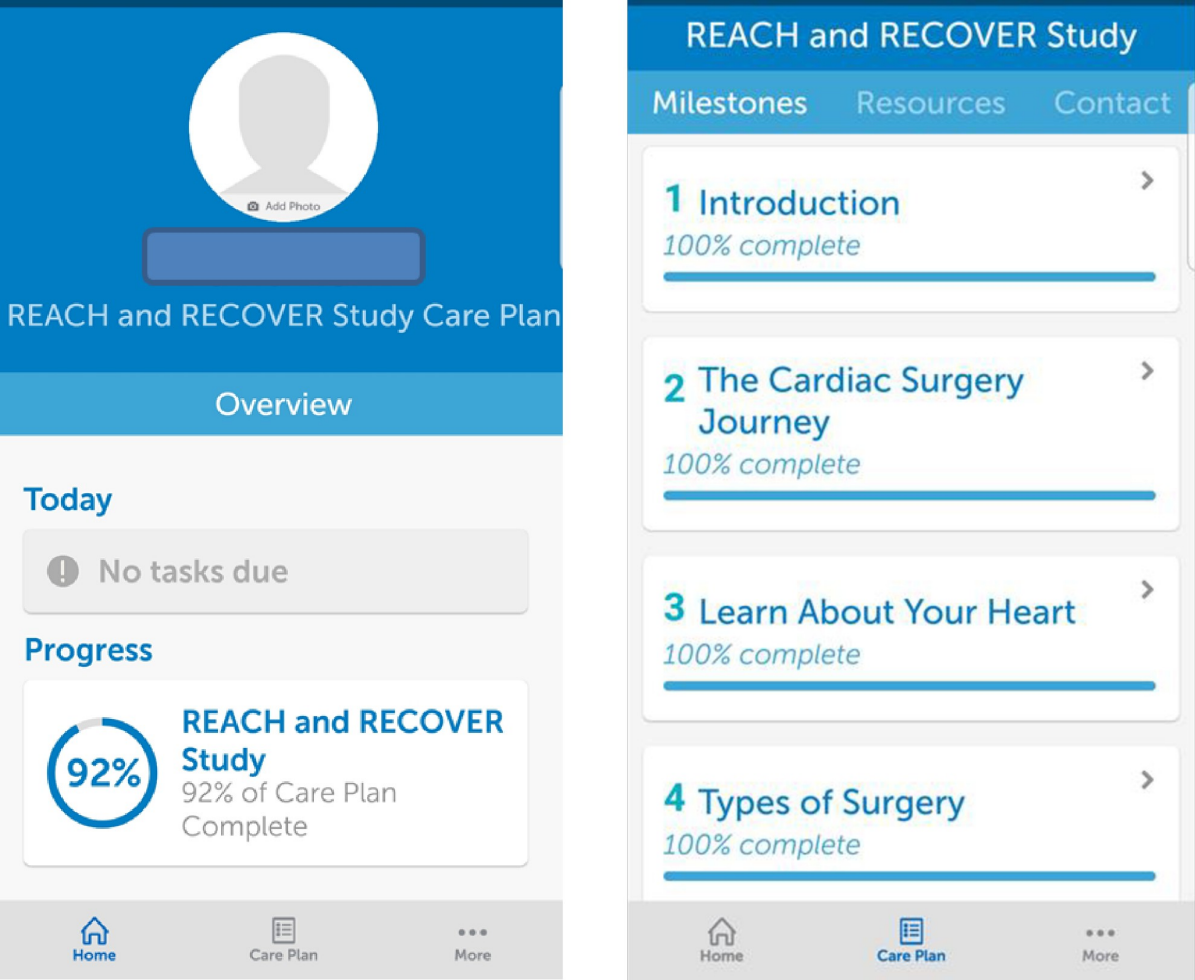
Screenshots of the mHealth Platform.

The mHealth app utilized in this study was developed by BeeWell Health. This software platform offers patient-facing patient care plans with various interactive features and functions. Using this pre-existing software as a starting framework, the content of the care plans was customized based on the patient engagement derived information and current cardiac educational material and ERPs implemented at St. Boniface Hospital. The app included three sections (Fig 2 and Appendix B in S1 Text):

1. Home page: provided user’s progress, today’s tasks, and day-to day reminders of overdue tasks which were updated on the app’s home page. Participants entered “anchor” dates of their pre-surgery assessment appointment and surgery date for the app to generate timed reminders for task completion. Upon pressing the specific tasks, participants were directed to a description of the task and the requirements to complete each one.
2. Care plan: this section was divided into 3 subsections:
  - Milestones: 13 items were included in this section regarding various phases of cardiac surgery journey. Written information and educational videos were included under each milestone.
  - Resources: Information regarding the cardiac surgery team, medications, and written and audiovisual educational material was provided in this section. Participants could also access information about hospital map, accommodation for out of town travellers and cardiac rehabilitation resources here.
3. Contact: Important phone numbers and links to email directory and hospital website was found here.

The content and functions of the app aimed to provide participants with comprehensive participant education, and to enhance patient engagement through timed reminders of daily health optimization goals pre- and postoperatively and tracking patient’s progress through the app. Enhancing patient education regarding their surgery allows patients to assume an active role in their care and improving adherence to ERPs guidelines [7].

During the app registration, participants were invited to create a profile on the app using their email address and password, which they had the opportunity to share with their caregivers to view the app on their own mobile devices. To best support health literacy among the varied cohort of participants, the app was designed with easy to understand language at grade seven level and was enhanced with educational videos and website links to online resources that participants could access. Participants were also provided with the regular (paper) St. Boniface educational booklet. Data regarding completed tasks (a surrogate for adherence) in the app were collected by the partnered technology company and analyzed by the research team.

### Self-reported measures

Participants completed four surveys at different time points of the study (Appendix A in S1 Text). The System Usability Survey (SUS) was administered to participants at the pre-surgery appointment and 6–8 weeks postoperatively. The survey gathered patients’ subjective assessment of the app’s usability. It contained 10 questions scored on a 5-point Likert scale, providing a final score ranging from 0–100 with higher scores indicating better usability [21]. Along with the SUS, participants completed a participant satisfaction survey developed specifically for this study by our research team. This survey gathered participants’ feedback regarding the content of the app for managing specific aspects of their care (e.g. taking medication, nutrition, and physical activity) and their acceptability of the app over written educational material. Participants also had the opportunity to provide comments regarding any items that they would add to the app to help them through the surgery process at the end of the participant satisfaction survey (Appendix C in S1 Text).

The EQ-5D-3L health-related quality of life (HRQoL) questionnaire is a standardized measure of health status. The questionnaire records participants’ self-reported assessments in the areas of mobility, self-care, usual activities, pain/discomfort and anxiety/depression. The EQ-VAS visual analogue scale assesses participants’ perception of their health (on a scale of 0–100) at the time of administration [23].

### Outcomes and statistical analysis

The primary outcome of this study is the feasibility (adherence, usability and user satisfaction) of the app. Adherence was defined by the completion rate of app tasks as collected by the partnered technology company. Secondary outcomes include 30-day hospital readmission obtained from electronic patient records, and HRQoL as measured by the EQ-5D-3L and EQ-VAS. Further analysis assessed the relationship between primary and secondary outcomes. A subgroup analysis was performed to detect differences in study outcomes between participants ≥ 65 and < 65 years of age.

Baseline characteristics of the study cohort were summarized using means and standard deviations for continuous variables and percentages for categorical variables. The characteristics of the EQ-5D-3L, Participant Satisfaction Survey, the System Usability Survey, and app adherence were summarized in a similar manner. These characteristics were also stratified by age (< 65 vs. ≥ 65 years) [18–20]. Spearman and point biserial correlations were calculated between the summary scores from the EQ-5D-3L and 30-day hospital readmission, and the Participant Satisfaction Survey, the System Usability Survey, and app adherence at the pre-surgery and post-discharge time points. All statistical analyses were performed using SAS version 9.4.

## Results

### Participant characteristics

**Fig 3:**
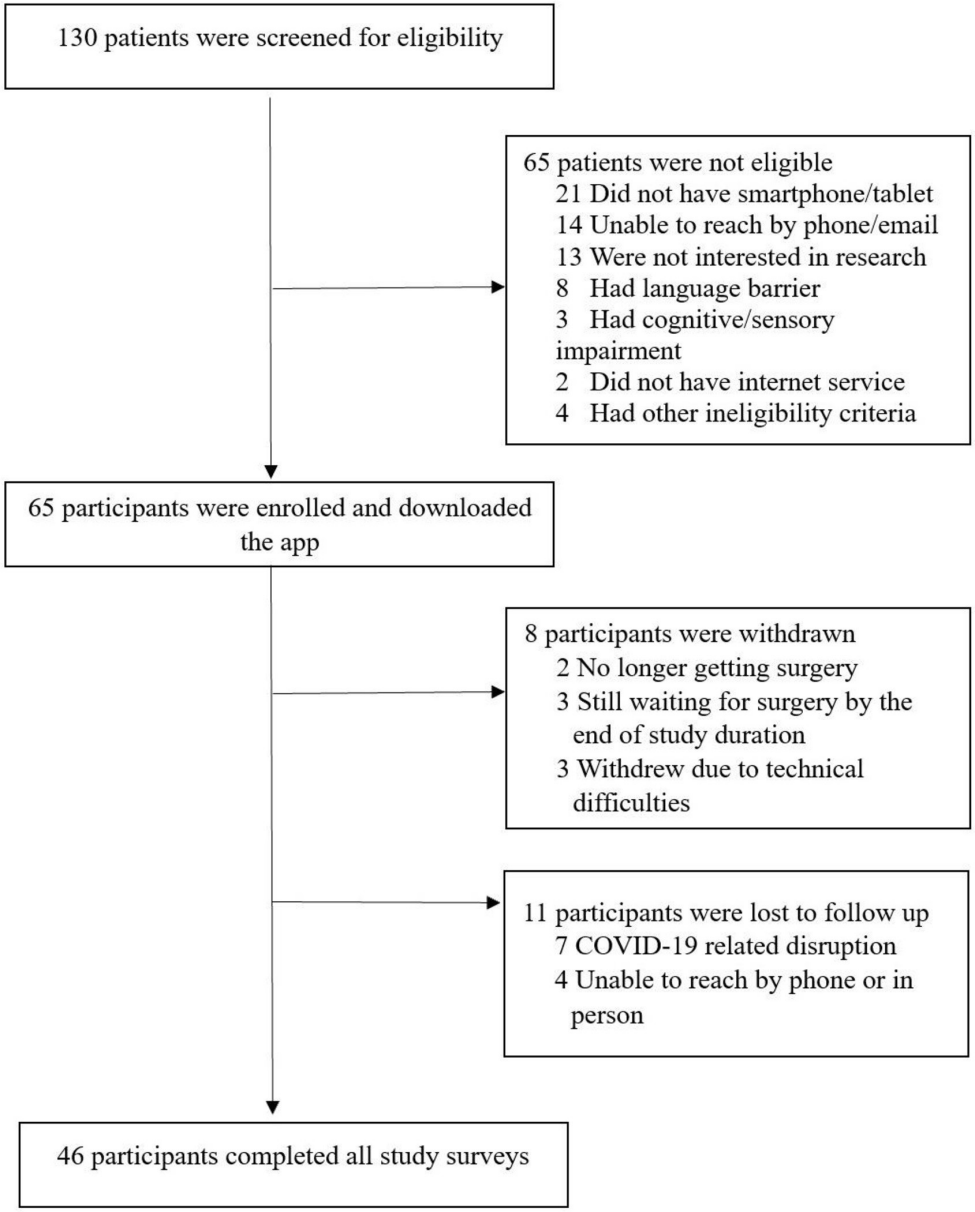
Participant Recruitment Flowchart.

A total of 130 participants were screened for study eligibility, and 65 consented to participate in the study (Fig 3). Of the 130 participants, 23 (18%) participants did not own a smartphone/tablet or internet access. After enrollment in the study, 8 participants were withdrawn (3 participants had technical difficulties and 5 withdrew consent) and 11 participants were lost to follow up. A total of 46 participants completed all pre- and post-surgery surveys. The mean age of participants was 64 years, with 26% of participants being female (n = 17). The most common procedure types were isolated valve surgery (n = 35, 54%) and isolated CABG (n = 19, 29%). Participant characteristics were captured and presented in Table 1. Appendix D in S1 Text outline participant characteristics stratified by completion status of study surveys.

**Table 1:** Characteristics of Participants (n = 65).

Characteristic	Study Cohort (n = 65)
Age in years, mean (SD)	64.3 (9.0)
Age ≥ 65 years, n (%)	33 (51)
Sex (Female), n (%)	17 (26)
**Comorbidities, n (%)**	
Hypertension	41 (63)
Dyslipidemia	36 (55)
Diabetes	18 (28)
COPD	5 (8)
Peripheral vascular Disease	0
Congestive Heart Failure	2 (3)
Previous Myocardial Infarction	2 (3)
Previous Cerebrovascular Accident	1 (2)
Atrial Fibrillation	9 (14)
**Ejection Fraction, n (%)**	
≥ 50%	51 (78)
35–49%	9 (14)
<35%	5 (8)
**Procedure Type, n (%)**	
Isolated CABG	19 (29)
Isolated Valve	43 (80)
CABG + Valve	3 (5)
**Operative Status, n (%)**	
Same Day Admission	60 (92)
Inpatient	5 (8)
**Hospital Disposition, n (%)**	
Died in Hospital	0
Discharged Home	57 (98)
Transferred to Anther Hospital	1 (2)
Hospital Length of Stay (Days)	7.6 (4.3)
30-Day Hospital Readmission	8 (12)

### mHealth adherence

Data regarding app usage was obtained for 57 participants with an overall task completion rate of 75%. The milestones with the highest completion rate included The Cardiac Surgery Journey (90%), Types of Surgery (83%), and Learn about Your Heart (82%). The milestones with the lowest completion rate included The Day You Leave the Hospital (66%), Recovering at Home (66%), and Cardiac Rehabilitation (62%). A higher percentage of participants ≥65 years completed the app tasks compared to participants <65 years (81% vs. 68%). The difference in completion rate was more prominent in the tasks related to the postoperative period, which achieved the lowest completion rate in the overall sample of participants (The Day You Leave the Hospital: 76% vs. 56%; Recovering at Home: 73% vs. 59%; Cardiac Rehabilitation: 71% vs. 53% for ≥65 and <65 years respectively) (Appendix E in S1 Text).

### mHealth usability

A total of 49 and 46 participants completed the SUS at pre-surgery and post-discharge, respectively. In the pre-surgery survey, over 90% of participants indicated that the app is easy to use (n = 46), were very confident using it (n = 45) and 89% found the app functions user-friendly (n = 44). Additionally, over 85% of participants disagreed with the need for a technical person to help them navigate the app (n = 43) or learning a lot before using the app (n = 42). Similarly, the majority of participants disagreed that the app is unnecessarily complex (n = 41, 83%), or too cumbersome to use (n = 43, 88%). A total of 37 (75%) participants reported that they would like to use the app frequently, which decreased to 57% in the post-discharge survey. The remaining responses did not change post-discharge (Appendix F in S1 Text). Further analysis by participant age revealed a lower agreement with app usability by participants ≥65 years compared to participants <65 years. A lower percentage of older participants agreed with the following questions compared to younger participants (presented as ≥65 vs. <65 years): would like to use the app frequently (42% vs. 73%), found the app unnecessarily complex (75% vs. 87%), too much inconsistency (84% vs. 96%), people would use the app quickly (79% vs. 91%), app is cumbersome to use (75% vs. 86%), and need to learn a lot before using the app (80% vs. 91%) (Appendix G in S1 Text).

### mHealth user satisfaction

A total of 50 and 46 participants completed the participant satisfaction survey at pre-surgery and post-discharge time points, respectively. Pre-surgery, 90% (n = 45) of participants found the educational information in the app useful, and 88% found the information easy to find (n = 44). Approximately half of the participants found the app helpful in managing their appointments (n = 25, 50%) and appreciated the reminders in the app (n = 27, 54%). Certain aspects of the app did not score highly in terms of participant satisfaction including managing medications (n = 10, 20%), nutrition (n = 15, 30%), and physical activity (n = 14, 28%). Overall, 62% (n = 31) of participants prefer the app over the written material and 90% (n = 45) would recommend the app. Post-discharge, 83% (n = 38) of the participants found the information in the app valuable to their recovery. Additionally, the satisfaction with the app to manage participants’ physical activity increased from pre-surgery to post-surgery (28% vs 54%). The remaining survey responses remained constant post-discharge (Appendix H in S1 Text).

More participants ≥65 years compared to <65 years agreed that the app helped them manage their appointments (58% vs. 45%), appreciated the reminders in the app (62% vs 50%), and liked inviting caregivers to the app (62% vs. 54%). A lower percentage of older participants (≥65 years) compared to younger participants (<65 years) indicated that they prefer the app over written information (51% vs. 87%) and that they would recommend the app (84% vs. 100%) in the post-surgery survey. The remaining responses were similar between the two age groups (Table 2).

**Table 2:** Post-Discharge MHealth Participant Satisfaction Responses by Age Category. Categorical variables expressed as n (%). Percentages include participants who Agreed or Strongly Agreed to survey questions.

Question	Age<65 (n = 22)	Age≥65 (n = 24)
**App Content**		
Education information useful to me	22 (100)	23 (96)
Help me manage my appointments	10 (45)	14 (58)
Help manage my meds	8 (37)	6 (25)
Help me manage my diet/nutrition	7 (32)	9 (38)
Help me manage my physical activity	11 (50)	14 (58)
Help me manage mental health	11 (50)	12 (50)
Info valuable to my recovery	20 (91)	18 (76)
**App Functions & Ease of Use**		
Appreciate reminders in app	11 (50)	15 (62)
Like inviting caregivers	12 (54)	15 (62)
Info easy to find	21 (95)	21 (88)
App difficult to use^a^	19 (86)	20 (83)
Took a long time to figure the app out^a^	20 (91)	21 (88)
**Overall Impression of App**		
Prefer app over written info	19 (87)	12 (51)
Would recommend the app	22 (100)	20 (84)

^a^ Score is assigned in reverse order for these questions

### Participants comments

Several participants commented on the comprehensive content of the app and the convenience and ease of having all the information, educational videos and website links in one place to access. This was important for participants to “feel prepared and at ease” and “reduce the anxiety” of undergoing major surgery. Participants commented on some of the aspects that could be improved in the app. Three participants commented on the need for more information on postoperative care, including normal progression of recovery, specific instructions on medication use, sternal wound dressing changes, and potential complications of the surgery. Two participants requested more information about types of replacement heart valve (to assist their decision-making process in choosing the appropriate valve). Enhanced calendar functionality to enter all appointment dates related to the surgery (such as cardiac rehabilitation appointments post-surgery) and allowing phone notifications, in addition to built-in app reminders, were also suggested by participants. Finally, a longer educational session at the time of app acquisition was recommended to optimize app navigation by participants. Textbox 1 provides a selection of quotes provided by participants regarding items that they would add to the app to help them through the surgery process (See Appendix I in S1 Text for all comments provided by participants).

**Textbox 1: Excerpts of Participants’ Responses Regarding the App**

“I liked the app since it helped reduce my anxiety regarding surgery” [Mr. D, 60 years]

“It was very informative + covered lots. Was good to have it and read it ahead and also go back to reread. Convenient!” [Mrs. A, 78 years]

“I needed more detailed walk-through using the app. Hoped [the app] was more lay terms in the app” [Mr. B, 58 years]

“I found the app based information much better to use than the papers” [Mr. D, 68 years]

“[Wanted] notification of the app on the phone” [Mr. D, 57 years]

“It had everything in there” [Mrs. L, 58 years]

“More information on post-op care,….,reminders were great” [Mr. F, 58 years]

“Perhaps a sample of daily calendar” [Mr. C, 53 years]

“Things to expect after discharge, “What is the normal progression?”. More information on post-op care info” [Mrs. VC, 81 years]

“Felt prepared and at ease because I watched and literally memorized the app” [Mr. K, 73 years]

“The best part was all the information, it was easy to find. I like that. More information is needed on choosing a mechanical vs tissue valve” [Mr. Y, 59 years]

“Detailed tutorial on where to find stuff in the app, would have liked more info for post-op care (i.e. info on sternal wound, medications etc.) Info about post-op complications (i.e., gout) would have been helpful” [Mrs. S, 67 years]

“I think it covered everything. But my app says only 20% completed” [Mr. M, 73 years]

### HRQoL and hospital readmission

At baseline, 25% (13/52) of participants reported some problems with mobility, which decreased to 4% (2/46) post-discharge. 33% (17/52) of participants reported moderate anxiety, decreasing to 13% (6/46) post-surgery. The percentage of participants reporting some pain appeared to remain the same despite undergoing surgery (29%, n = 15 vs. 26%, n = 12, at baseline and post-discharge, respectively). The mean EQ-Visual score was 70.2 at baseline and 79 post-discharge (Appendix J in S1 Text). After undergoing surgery, 98% of participants were discharged home, with eight participants (12%) experiencing a 30-day hospital readmission (Table 3). Pre-surgery, a higher score on the participant satisfaction survey was associated with a higher EQ-Visual score. No other associations were observed between primary and secondary outcomes of the pre-surgery and post-discharge surveys (Table 3).

**Table 3:** Association Between App Feasibility and Quality of Life and 30-Day Hospital Readmission.

Time Period: Pre-Surgery^a^		
Characteristic	EQ-Visual Analog Scale*	30 Day hospital Readmission
	Spearman Correlation	Point Biserial Correlation
Participation Satisfaction Survey (Total Score) ^a^	0.445	0.150
System Usability Survey (Total Score) ^a^	0.236	0.194
App Adherence (Total Score)	0.004	0.002
**Time Period: Post-Discharge** ^b^		
**Characteristic**	EQ-Visual Analog Scale**	**30 Day Rehospitalization**
	**Spearman Correlation**	**Point Biserial Correlation**
Participation Satisfaction Survey (Total Score) ^b^	0.059	0.079
System Usability Survey (Total Score) ^b^	-0.183	-0.044
App Adherence (Total Score)	0.152	0.002

^a^ Pre-surgery

^b^ Post-discharge

## Discussion

### Principle findings

This prospective cohort study is one of the first to evaluate the use of mHealth for peri-operative CS patient education. Previous research in this area focused primarily on postoperative patient care [16,21,22,27] with the employment of telemedicine [28], rather than providing comprehensive peri-operative patient education using an app-based platform. The majority of enrolled participants reported high satisfaction with the app and rated the app highly in terms of its usability. The mHealth app employed in this study utilized content developed by the CS team based on evidence-based ERPs that were customized to the local clinical setting. Additionally, the engagement of patients in the development of the app was particularly important, given that patients expressed their desire to be included in the app design and development processes [29] and the importance of patient engagement in research [23], including mHealth technology design [24]. Inviting caregivers to share app use with patients is helpful to assist inexperienced patients with app navigation if needed and improve their overall knowledge of the CS journey to better support the patient’s recovery [30].

The app used for this initiative achieved a task completion rate of 75%, with a 5% attrition rate due to technical difficulties. These are promising results given that adherence to mHealth app usage has been commonly cited as a major hindrance to the integration of such technologies in patient care [31]. Previous studies reported low adherence to app usage and high attrition rate mostly due to technological difficulties [32], and lack of interest in technology uptake [33]. In our study, there was a gradual decrease in app usage over time, with post-discharge tasks achieving the lowest completion rate. This pattern was similarly demonstrated in other studies [34–37]. This decline in app usage during the post-operative period could be attributed to the long duration between app teaching at the time of consent and surgery date, and the patients’ direct contact with the treating team in hospital at the time of surgery. Education reinforcement by the medical team at the time of discharge could be beneficial to increase usability of the app post-discharge. Despite a gradual decrease in our app usage through its content, the lowest completed task was accessed by 62% of participants. In fact, the completion rate presented in the study could be underestimated with our current methodology, given that participants had to click the “Complete Task” button at the bottom of each task for the task to be considered complete. This was the case for one of the participants who mentioned that they completed all tasks, but the app reported 20% completion rate. The high adherence rate in our study could be attributed to the simplicity of the app, a crucial feature for mHealth uptake [38,39]. This is supported by the majority of participants agreeing that the app was easy to use and their confidence in using it without assistance.

Several concerns have been raised regarding the use of mHealth in older adults. Specifically, the slow uptake of mHealth in older adults has been attributed to technical difficulties, low self-efficacy to operate devices, and poor perception of app usefulness [29] Despite having a similar percentage of participants between the two age groups (≥65 years and <65 years) disagreeing with the difficulty of app usage and the need for a technical person to help them navigate the app, as well as an overall higher task completion rate by older participants, more participants ≥65 years indicated their preference for written material over the app. This could speak to the unfamiliarity with app-based technologies in general in this age group rather than technical illiteracy hindering app use. As some participants recommended, a longer educational session to familiarize patients with the app could be helpful to increase older patients acceptability of mHealth for patient education [40] The design of the app could also be refined specifically for older patients through additional patient engagement activities carried out with older adult patients [24].

Certain survey questions did not receive favourable rating from participants. The low satisfaction in the domains of medication management, physical activity, and nutrition could be attributed to multiple factors. First, some participants expressed their limited physical abilities as a barrier to perform physical activity, which was inaccurately reflected on the app’s usefulness in the survey responses. Second, the app provided general information about commonly used medications and the physical activity and nutritional requirements, rather than a personalized plan for each patient. Personalization of app content has been shown to highly affect behavioural change in patients [25,29]. However, complex medical comorbidities in the CS patient population and the various peri-operative considerations that are unique to each patient preclude the customization of ERPs to each patient [41]. Additionally, the decrease in the number of patients that would like to use the app frequently in the post-surgery survey could be due to the reduced need to review educational material regarding the surgery as patient’s recovery progressed, rather than diminishing interest in app usage and usefulness after surgery.

The feasibility of the mHealth intervention in this study was not associated with an improvement in HRQoL, however this was not the primary goal of this initiative at this stage. The reduction in anxiety and improvement in mobility and mean EQ-Visual score is likely attributed to undergoing the surgery and improvement in health independent of app usage. Previous systematic reviews regarding mHealth interventions indicated an improvement in quality of life using telehealth interventions that facilitated patient-provider interaction and targeted symptom management specifically [33,42], whereas the mHealth app used in our study targets the domain of patient education and intends to replace paper-based patient education. Moreover, the small sample size in this study may have impacted the ability to detect subtle correlations between mHealth usage and quality of life improvement. Similarly, the hospital readmission rate found in this study is in line with previous studies conducted at St. Boniface Hospital [33,40,42,43]. The rate of hospitalization in Manitoba, however, is lower than other Canadian jurisdictions [44], which could have potentially affected the ability to detect an association between app usage and hospital readmission rates.

This study has several strengths. The intervention employed in this study offered various benefits to patients, as discussed previously. In addition, there has been increasing recognition of the importance of patient-reported outcomes (PROs) such as quality of life besides traditionally measured objective outcomes. Also, PROs provide a more accurate capture of patient experience as opposed to clinician-performed assessments [45]. A subgroup analysis was performed to evaluate the feasibility of mHealth by patient age, with all CS patients recruited in the study regardless of age. Adherence to app usage was measured through the app, providing an objective and accurate assessment, rather than relying on self-reported questionnaires [43].

## Limitations

There are also limitations to the study to be acknowledged. This study was conducted in a single clinical setting with a relatively small sample size, affecting the generalizability of study results. Participant recruitment based on a volunteer basis is prone to selection bias. Patients who agreed to participate were required to have access to a device or internet connection and may have been more likely to be familiar with mobile technology and motivated to use the app. A significant number of screened participants (18%) were unable to participate in the study due to a lack of cellphone/internet access. A healthcare system provided tablet could, in theory, be used by patients in the future for the duration of their peri-operative period [46]. Of note, internet access is not required for app use, but rather to track app usage data for study purposes only. Additionally, about 10% of screened patients were not interested in research; feeling overwhelmed by the CS experience was the most reported reason for study participation decline, which is similar to previous studies [16]. This study did not assess the impact of patient education through mHealth on behavioural change, such as medication adherence and meeting exercise goals. Future studies will aim to evaluate clinical outcomes after mHealth technology implementation.

## Conclusion

The use of mHealth technology for peri-operative patient education is feasible to implement in the CS patient population, including older adult patients. The majority of enrolled patients were satisfied with the app and would recommend it over the use of printed materials. In the future, additional studies should be undertaken to determine if the use of mHealth technology results in behavioural change and increased adherence to ERPs protocols as well as other important patient-centred outcomes.

## Supporting information

S1 Text Appendix A. Study Timeline. Appendix B. Screenshots of the mHealth Platform. Appendix C. Participant Satisfaction Survey (App). Appendix D. Characteristics of Participants (n = 65) stratified by survey completion status. Appendix E. mHealth Adherence by Age Group. Appendix F. mHealth Usability Reported by Participants. Appendix G. Post-Discharge mHealth Usability by Age Group. Appendix H. MHealth Participant Satisfaction Responses. Appendix I. Participant Comments Regarding the App. Appendix J. Self-Reported Health-Related Quality of Life.
(DOCX)
