# Supplementary material for: Use of a mobile health application by adult non-congenital cardiac surgery patients: A feasibility study
Source: PLOS Digit Health. 2022 Jun 29;1(6):e0000055. doi: 10.1371/journal.pdig.0000055 (PMC9931304; doi:10.1371/journal.pdig.0000055)
Supplement: S1 Text — Appendix A. Study Timeline. Appendix B. Screenshots of the mHealth Platform. Appendix C. Participant Satisfaction Survey (App). Appendix D. Characteristics of Participants (n = 65) stratified by survey completion status. Appendix E. mHealth Adherence by Age Group. Appendix F. mHealth Usability Reported by Participants. Appendix G. Post-Discharge mHealth Usability by Age Group. Appendix H. MHealth Participant Satisfaction Responses. Appendix I. Participant Comments Regarding the App. Appendix J. Self-Reported Health-Related Quality of Life. (DOCX) [file pdig.0000055.s001.docx]

**Appendix A.** Study Timeline

**Baseline**

- EQ-5D-3L
- EQ-VAS
- System Usability Survey
- Patient Satisfaction Survey
- EQ-5D-3L
- EQ-VAS
- EQ-5D-3L
- EQ-VAS

**Pre-discharge**

- EQ-5D-3L
- EQ-VAS
- System Usability Survey
- Patient Satisfaction Survey

**Pre-operative appointment**

**6-8 weeks post-discharge**

**Appendix B.** Screenshots of the mHealth Platform


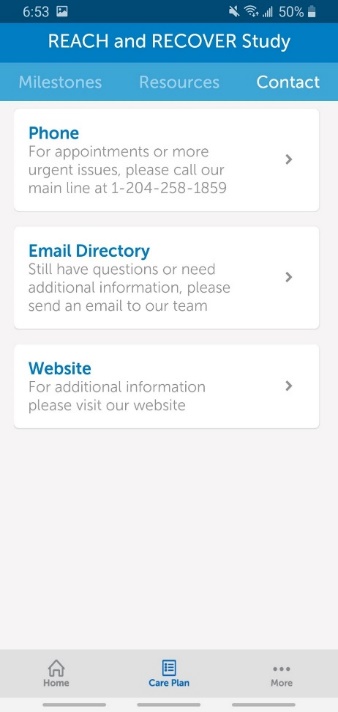

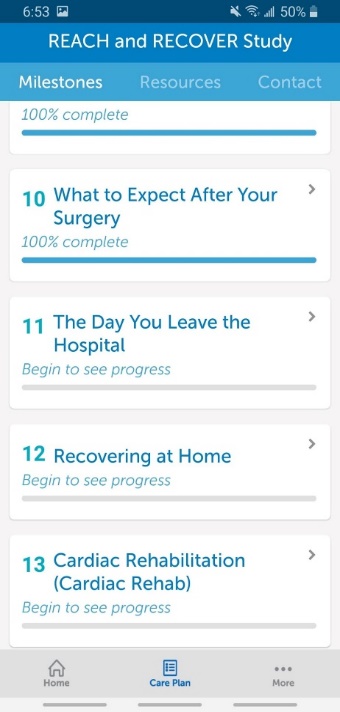

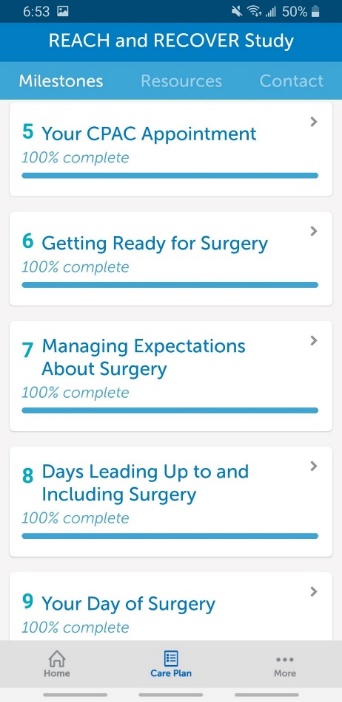

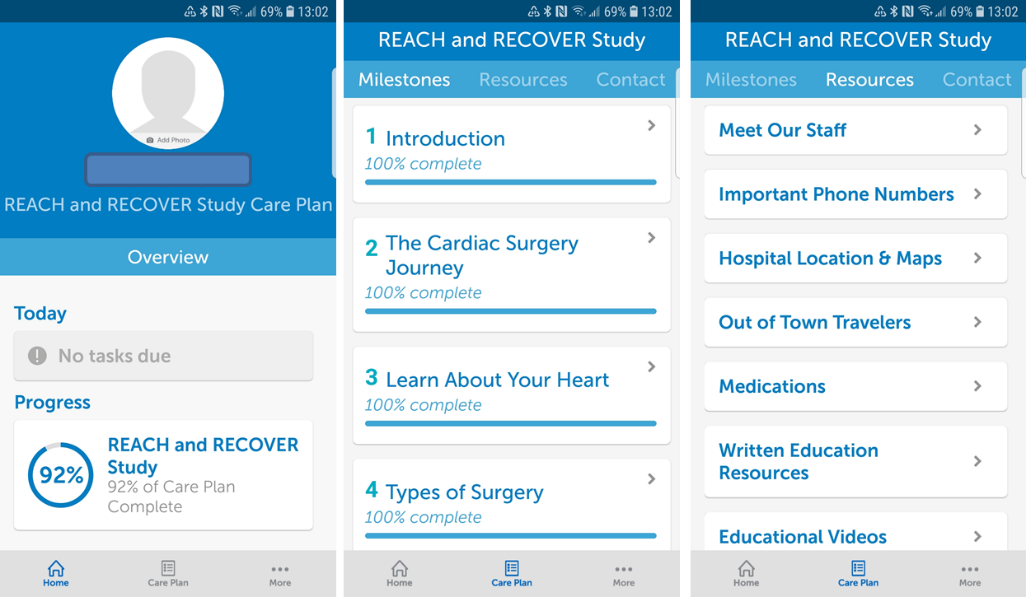

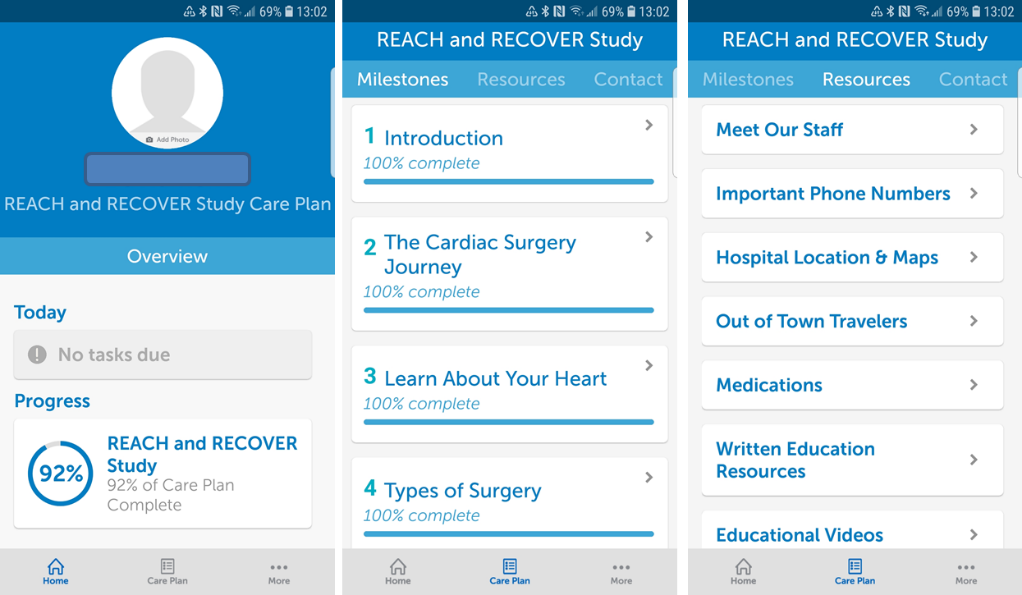

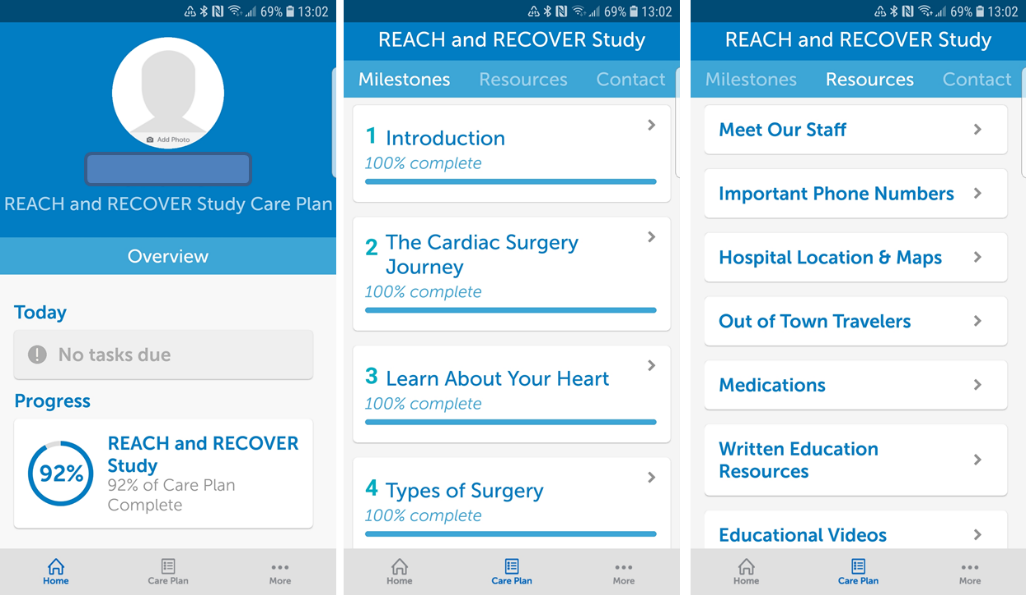


**Appendix C.** Participant Satisfaction Survey (App)

| **Questions** | **Strongly Disagree** | **Disagree** | **Neutral** | **Agree** | **Strongly Agree** |
| --- | --- | --- | --- | --- | --- |
| 1) I found the education information included in the App was useful to me | 1 | 2 | 3 | 4 | 5 |
| 2) I found the app was useful in helping me to manage my medical appointments | 1 | 2 | 3 | 4 | 5 |
| 3) I found the app was useful in helping me to manage taking my medications | 1 | 2 | 3 | 4 | 5 |
| 4) I found the app was useful in helping me to manage my diet/nutrition | 1 | 2 | 3 | 4 | 5 |
| 5) I found the app was useful in helping me to manage my physical activity | 1 | 2 | 3 | 4 | 5 |
| 6) I found the app was useful in helping me to manage my mental health (i.e. stress, anxiety) | 1 | 2 | 3 | 4 | 5 |
| 7) I found the information on the App was easy to find | 1 | 2 | 3 | 4 | 5 |
| 8) I found the information provided on the App was valuable to my recovery | 1 | 2 | 3 | 4 | 5 |
| 9) I prefer the App based information over the written information provided to me | 1 | 2 | 3 | 4 | 5 |
| **Questions** | **Strongly Disagree** | **Disagree** | **Neutral** | **Agree** | **Strongly Agree** |
| 10) I found the App very difficult to use while looking for the information I needed (r) | 1 | 2 | 3 | 4 | 5 |
| 11) It took me a long time to figure the App out (r) | 1 | 2 | 3 | 4 | 5 |
| 12) I really appreciated the reminders the App provided for me | 1 | 2 | 3 | 4 | 5 |
| 13) I liked how I was able to invite my care-giver(s) to the App to view the surgery process | 1 | 2 | 3 | 4 | 5 |
| 14) I would recommend this App to other cardiac surgery patients | 1 | 2 | 3 | 4 | 5 |

Are there other items that you would add to this App to help you through the surgery process?

Yes No

If yes, please take some time and list your comments below.

Thank you!

**Scoring Personal Satisfaction survey**

Survey is classified into 2 sections. The scores will be added separately when scoring questionnaire.

Forward score (questions 1-9 and 12-14) (Scale -1)

Max score = 48

Minimal Score = 0

Reverse score (Questions 10 and 11) (5 – Scale)

Max score = 8

Minimal score = 0

Total Score Calculation

- To calculate the PSS score, first sum the score contributions from each item (Each item's score contribution will range from 0 to 4. For items 1- 9 and 12-14 the score contribution is the scale position minus 1. For items 10 and 11, the contribution is 5 minus the scale position).
- Multiply the sum of the scores by 1.785 to obtain the overall value of PSS.

PSS scores have a range of 0 to 100.

**Appendix D.** Characteristics of Participants (n=65) Stratified by Survey Completion Status.

| **Characteristic** | | **Did not Respond at Post-Discharge (n=19)** | **Responded at Post-Discharge (n=46)** |
| --- | --- | --- | --- |
| Age in years, mean (SD) | | 62.6 (9.0) | 65.0 (8.9) |
| Age ≥ 65 years, n (%) | | 9 (47) | 24 (52) |
| Sex (Female), n (%) | | 6 (32) | 11 (24) |
| **Comorbidities, n (%)** | |  |  |
|  | Hypertension | 13 (68) | 28 (61) |
|  | Dyslipidemia | 9 (47) | 27 (59) |
|  | Diabetes | 3 (16) | 15 (33) |
|  | COPD | 2 (11) | 3 (7) |
|  | Peripheral vascular Disease | 0 (0) | 0 (0) |
|  | Congestive Heart Failure | 0 (0) | 2 (4) |
|  | Previous Myocardial Infarction | 0 (0) | 2 (4) |
|  | Previous Cerebrovascular Accident | 1 (5) | 0 (0) |
|  | Atrial Fibrillation | 0 (0) | 9 (20) |
| **Ejection Fraction, n (%)** | |  |  |
|  | ≥ 50% | 16 (84) | 35 (76) |
|  | 35-49% | 2 (11) | 7 (15) |
|  | <35% | 1 (5) | 4 (9) |
| **Procedure Type, n (%)** | |  |  |
|  | Isolated CABG | 3 (16) | 16 (35) |
|  | Isolated Valve | 13 (68) | 22 (48) |
|  | CABG + Valve | 0 (0) | 1 (2) |
| **Operative Status, n (%)** | |  |  |
|  | Same Day Admission | 16 (84) | 43 (93) |
|  | Inpatient | 2 (11) | 3 (6) |
| **Hospital Disposition, n (%)** | |  |  |
|  | Died in Hospital | 0 (0) | 0 (0) |
|  | Discharged Home | 19 (100) | 45 (98) |
|  | Transferred to Anther Hospital | 0 (0) | 1 (2) |
|  | Hospital Length of Stay (Days) | 8.8 (4.4) | 7.2 (4.2) |
|  | 30-Day Hospital Readmission | 2 (17) | 6 (13) |

**Appendix E.** mHealth Adherence by Age Group**.**

Categorical variables expressed as N (%).

| **Milestone** |  |  |  |
| --- | --- | --- | --- |
|  | **All Participants (N=57)** | **Age<65 (N=29)** | **Age≥65 (N=28)** |
| Introduction | 54 (95) | 26 (91) | 28 (100) |
| The Cardiac Surgery Journey | 51 (90) | 26 (90) | 25 (91) |
| Learn About Your Heart | 47 (82) | 22 (76) | 25 (89) |
| Types of Surgery | 47 (83) | 23 (78) | 25 (88) |
| Your CPAC Appointment | 41 (79) | 21 (72) | 24 (86) |
| Getting Ready for Surgery | 45 (79) | 21 (73) | 24 (86) |
| Managing Expectations About Surgery | 43 (75) | 21 (72) | 22 (79) |
| Days Leading Up to and Including Surgery | 42 (74) | 20 (70) | 22 (79) |
| Your Day of Surgery | 42 (74) | 20 (69) | 22 (79) |
| What to Expect After Your Surgery | 42 (74) | 19 (67) | 23 (82) |
| The Day You Leave the Hospital | 38 (66) | 16 (56) | 22 (76) |
| Recovering at Home | 38 (66) | 17 (59) | 21 (73) |
| Cardiac Rehabilitation (Cardiac Rehab) | 35 (62) | 15 (53) | 20 (71) |
| Overall App Utilization (%) | 74.5 | 68.3 | 81 |

**Appendix F.** mHealth Usability Reported by Participants.

Categorical variables expressed as N (%) and continuous variables expressed as mean (standard deviation). P-values calculated using paired T-test for individuals who responded at both time points.

| **Question** | **Pre-Surgery (N=49)** | | | | | | **Post-Discharge (N=46)** | | | | | | | | |  |  |  |
| --- | --- | --- | --- | --- | --- | --- | --- | --- | --- | --- | --- | --- | --- | --- | --- | --- | --- | --- |
|  | **Strongly Disagree (0)/ Disagree (1)** | **Neutral (2)** | **Agree (3)/ Strongly Agree (4)** | | | **Score (/4)** | **Strongly Disagree (0)/ Disagree (1)** | | | | **Neutral (2)** | **Agree (3)/ Strongly Agree (4)** | | **Score**  **(/4)** | |  |  |  |
| 1) Would like to use app frequently | 3 (6) | 9 (18) | 37 (75) | | | 2.8 (0.7) | 12 (26) | | | | 8 (17) | 26 (57) | | 2.4 (1.0) | |  |  |  |
| 2) Found app unnecessarily complex* | 41 (83) | 5 (10) | 3 (4) | | | 3.0 (0.8) | 37 (81) | | | | 4 (9) | 5 (11) | | 2.8 (0.8) | |  |  |  |
| 3) App easy to use | 2 (4) | 1 (2) | 46 (94) | | | 3.2 (0.7) | 2 (4) | | | | 5 (11) | 39 (85) | | 3.0 (0.7) | |  |  |  |
| 4) Would need technical person to help* | 43 (88) | 2 (4) | 4 (8) | | | 3.2 (0.9) | 41 (89) | | | | 2 (4) | 3 (6) | | 3.0 (0.8) | |  |  |  |
| 5) Functions in app were user-friendly | 2 (4) | 3 (6) | 44 (89) | | | 3.0 (0.8) | 2 (4) | | | | 6 (13) | 38 (83) | | 2.8 (0.7) | |  |  |  |
| 6) Too much inconsistency* | 41 (83) | 4 (8) | 4 (8) | | | 3.0 (0.8) | 41 (89) | | | | 3 (7) | 2 (4) | | 3.0 (0.6) | |  |  |  |
| 7) People would use app quickly | 3 (6) | 6 (12) | 40 (81) | | | 3.0 (0.8) | 0 | | | | 7 (15) | 39 (85) | | 2.9 (0.5) | |  |  |  |
| 8) App cumbersome to use* | 43 (88) | 2 (4) | 4 (8) | | | 3.1 (0.9) | 37 (80) | | | | 6 (13) | 3 (7) | | 2.9 (0.7) | |  |  |  |
| 9) Very confident using app | 2 (4) | 2 (4) | 45 (92) | | | 3.2 (0.8) | 2 (4) | | | | 4 (9) | 40 (87) | | 3.0 (0.7) | |  |  |  |
| 10) Need to learn a lot before using app* | 42 (86) | 2 (4) | 5 (10) | | | 3.0 (0.9) | 39 (85) | | | | 4 (9) | 3 (6) | | 2.9 (0.8) | |  |  |  |
| Total Standardized Score (/100) |  |  |  | | | 76.1 (16.5) |  | | | |  |  | | 71.7 (15.3) | |  |  |  |
| *Score is assigned in reverse order for these questions | | |  |  |  | | |  |  |  | | |  | |  | |  |  |

**Appendix G.** Post-Discharge mHealth Usability by Age.

Categorical variables expressed as N (%). Percentages include participants who Agreed or Strongly Agreed to survey questions.

| **Question** | **Age<65 (N=22)** | **Age≥65 (N=24)** |
| --- | --- | --- |
| 1) Would like to use app frequently | 16 (73) | 10 (42) |
| 2) Found app unnecessarily complex^a^ | 19 (87) | 18 (75) |
| 3) App easy to use | 19 (87) | 20 (84) |
| 4) Would need technical person to help^a^ | 20 (91) | 21 (88) |
| 5) Functions in app were user-friendly | 19 (86) | 19 (79) |
| 6) Too much inconsistency^a^ | 21 (96) | 20 (84) |
| 7) People would use app quickly | 20 (91) | 19 (79) |
| 8) App cumbersome to use^a^ | 19 (86) | 18 (75) |
| 9) Very confident using app | 20 (91) | 20 (84) |
| 10) Need to learn a lot before using app^a^ | 20 (91) | 19 (80) |

^a^Score is assigned in reverse order for these questions

**Appendix H.** MHealth Participant Satisfaction Responses.

Categorical variables expressed as N (%) and continuous variables expressed as mean (standard deviation). P-values calculated using paired T-test for individuals who responded at both time points.

| **Question** | **Pre-Surgery (N=50)** | | | | | | |  |  |  |  |  |  |  |  |  |  |  |  |  |  |  |
| --- | --- | --- | --- | --- | --- | --- | --- | --- | --- | --- | --- | --- | --- | --- | --- | --- | --- | --- | --- | --- | --- | --- |
|  | **Strongly Disagree (0)/ Disagree (1)** | **Neutral (2)** | | **Agree (3)/ Strongly Agree (4)** | | | **Score** | **Strongly Disagree (0)/ Disagree (1)** | **Neutral (2)** | | **Agree (3)/ Strongly Agree (4)** | | | | **Score** | | |  |  |  |  |  |
| 1) Education information useful to me | 0 (0) | 5 (10) | | 45 (90) | | | 3.3 (0.6) | 1 (2) | 0 | | 45 (98) | | | | 3.2 (0.5) | | |  |  |  |  |  |
| 2) Help me manage my appointments | 7 (14) | 18 (36) | | 25 (50) | | | 2.5 (0.9) | 18 (39) | 4 (9) | | 24 (62) | | | | 2.2 (1.1) | | |  |  |  |  |  |
| 3) Help manage my medications | 13 (26) | 27 (54) | | 10 (20) | | | 1.9 (1.0) | 23 (50) | 9 (20) | | 14 (31) | | | | 1.8 (1.0) | | |  |  |  |  |  |
| 4) Help me manage my diet/nutrition | 9 (18) | 26 (52) | | 15 (30) | | | 2.2 (0.9) | 19 (41) | 11 (24) | | 16 (35) | | | | 2.0 (1.0) | | |  |  |  |  |  |
| 5) Help me manage my physical activity | 11 (22) | 25 (50) | | 14 (28) | | | 2.1 (0.9) | 11 (24) | 10 (22) | | 25 (54) | | | | 2.4 (1.0) | | |  |  |  |  |  |
| 6) Help me manage mental health | 7 (14) | 17 (34) | | 26 (52) | | | 2.5 (1.0) | 12 (26) | 11 (24) | | 23 (50) | | | | 2.3 (0.9) | | |  |  |  |  |  |
| 7) Info easy to find | 2 (4) | 4 (8) | | 44 (88) | | | 3.2 (0.7) | 2 (4) | 2 (4) | | 42 (92) | | | | 3.1 (0.7) | | |  |  |  |  |  |
| 8) Info valuable to my recovery | 0 (0) | 34 (68) | | 16 (32) | | | 2.5 (0.7) | 5 (11) | 3 (7) | | 38 (83) | | | | 2.9 (0.9) | | |  |  |  |  |  |
| 9) Prefer app over written info | 3 (6) | 16 (32) | | 31 (62) | | | 2.9 (0.9) | 6 (13) | 9 (20) | | 23 (68) | | | | 2.8 (0.9) | | |  |  |  |  |  |
| 10) App difficult to use* | 40 (80) | 6 (12) | | 4 (8) | | | 2.9 (0.8) | 39 (85) | 4 (9) | | 3 (7) | | | | 2.9 (0.7) | | |  |  |  |  |  |
| 11) Took a long time to figure the app out* | 43 (86) | 4 (8) | | 3 (6) | | | 3.1 (0.8) | 41 (89) | 2 (4) | | 3 (7) | | | | 3.0 (0.7) | | |  |  |  |  |  |
| 12) Appreciate reminders in app | 3 (6) | 20 (40) | | 27 (54) | | | 2.6 (0.8) | 10 (22) | 10 (22) | | 26 (57) | | | | 2.4 (0.9) | | |  |  |  |  |  |
| 13) Like inviting caregivers | 2 (4) | 24 (48) | | 24 (48) | | | 2.5 (0.7) | 8 (17) | 11 (24) | | 27 (59) | | | | 2.5 (0.9) | | |  |  |  |  |  |
| 14) Would recommend the app | 1 (2) | 4 (8) | | 45 (90) | | | 3.4 (0.7) | 1 (2) | 3 (7) | | 42 (91) | | | | 3.4 (0.7) | | |  |  |  |  |  |
| Total Standardized Score (/100) |  |  | |  | | | 66.9 (13.0) |  |  | |  | | | | 66.1 (14.4) | | |  |  |  |  |  |
| *Score is assigned in reverse order for these questions | | |  | |  |  | | | |  | |  |  |  | |  |  | |  |  |  |  |

**Appendix I.** Participant Comments Regarding the App and What They Would Like to See Added to the App.

| “I liked the app since it helped reduce my anxiety regarding surgery. The app does not help with nutrition or physical activity since there is no information about that pre-procedure, Videos in the app are informative, but old. [Wants to see] scheduling, more reminders, CT scan and angiogram appointments ” [Mr. D, 60 years]  “It was very informative + covered lots. Was good to have it and read it ahead and also go back to reread. Convenient!” [Mrs. A, 78 years]  “ Realistic timetable regarding surgery date (as participant has been getting conflicting information regarding potential surgery date from surgeon and staff so he was hoping for a function of the app to facilitate monitoring his progress on the surgery waitlist)” [Mr. H, 76 years]  “Was wondering if app can be provided to patients at time of referral from cardiology to cardiac surgery, explained app intended for patients 100% going for surgery (not just referred to cardiac surgery). Wants to see dates entered + reminders about tasks for Cardiac Rehab appointment” [Mr. B, 64 years]  “Patient was wondering if we can transfer the app on USB to use it on desktop” [Mr. C, 57 years]  “Information not accurate (timeframes, time efore surgery), Active notifications” [Mr. H, 67 years]  “I needed more detailed walk-through using the app. Hoped [the app] was more lay terms in the app. Heart and Stroke book on rehab- a link to PDF would be helpful” [Mr. B, 58 years]  “I don’t think it was programmed well for the caregivers", "I did not find the app helpful at all", Ottawa heart book online was useful more than St. Boniface.” [Mr. B, 72 years]  “Information on bathing” [Mr. G, 71 years]  “ Surgery date confusions are frustrating” [Mr. L, 71 years]  “I found the app based information much better to use than the papers” [Mr. D, 68 years]  “[Wanted] notification of the app on the phone” [Mr. D, 57 years]  “It had everything in there” [Mrs. L, 58 years]  “ App did not flow with the cardiac surgery schedule. Very convenient” [Mr. D, 61 years]  “[Wanted detailed tutorial on where to find stuff in the app , would have liked more info for post-op care (i.e. info on sternal wound, medications etc.). Nobody tells you how long you will be on medications for. Info about post-op complications (i.e. gout) would have been helpful” [Mrs. S, 67]  “The best part was all the info, it was easy to find. I like that. More info is needed on choosing mechanical vs. tissue valve” [Mr. Y, 59 years]  “Increase information on valves - a lot of info on coronary - have them separate - didn’t need to see the coronary info. "Your heart (error) heart valves act like doors. Was not able to change appointment dates once in the app. Frustrating” [Mr. S, 59 years]  “ Bias in the app-if you have heart disease it is your fault. Felt prepared and at ease because I watched and literally memorized the app” [Mr. K, 73 years]  “Overall the app tended to focus on depression/ anxiety. Needs to also present a positive outlook. The Bypass operation is major, BUT gives people a new lease on life - VERY POSITIVE/ IMPORTANT. I told my 40 year old sons they would now have trouble once i full recover! I also advised my wife she will have to get into better shape to keep up. I have been working 4 days/week and will cut back further, to enjoy living and travelling more” [Mr. S, 70]  “Remind them about over worrying is common and teach how to deal” [Mr. B, 57]  “More information on post-op care, video quality was poor, reminders were great” [Mr. F, 58 years]  “Perhaps a sample of daily calendar. Better videos, better post-op information” [Mr. C, 53 years]  “Things to expect after discharge, “What is the normal progression?”. More information on opst-op care info” [Mrs. VC, 81 years]  “I think it covered everything. But my app says only 20% completed” [Mr. M, 73 years]  “I deleted the app 3 months ago because I did not really use it after surgery” [Mrs. M, 72 years]  “More pictures and diagrams-sometimes pictures speak a 1000 words” [Mr. G, 79 years]  “Add a bit on valve repair. [Patient] had bypass and valve replacement” [Mr. S, 53 years] |
| --- |

**Appendix J.** Health-Related Quality of Life Reported by Participants.

Categorical variables expressed as N (%). Continuous variables expressed as mean (standard deviation).

| **Characteristic** | **Baseline (N=52)** | **Pre-Surgery (N=51)** | **Pre-Discharge (N=43)** | **Post-Discharge (N=46)** |  |
| --- | --- | --- | --- | --- | --- |
| **Mobility** |  |  |  |  | |
| Some Problems | 13 (25) | 13 (25) | 11 (26) | 2 (4) | |
| Confined to Bed | 0 | 1 (2) | 1 (2) | 0 | |
| **Self Care** |  |  |  |  | |
| Some Problems | 2 (4) | 5 (10) | 13 (30) | 2 (4) | |
| Unable to Perform | 0 | 1 (2) | 4 (9) | 0 | |
| **Usual Activities** |  |  |  |  | |
| Some Problems | 21 (40) | 17 (33) | 28 (65) | 13 (28) | |
| Unable to Perform | 2 (4) | 4 (8) | 4 (9) | 1 (2) | |
| **Pain/Discomfort** |  |  |  |  | |
| Some Pain | 15 (29) | 26 (51) | 26 (60) | 12 (26) | |
| Extreme Pain | 0 | 0 | 3 (7) | 0 | |
| **Anxiety/Depression** |  |  |  |  | |
| Moderate | 17 (33) | 24 (47) | 9 (21) | 6 (13) | |
| Extreme | 2 (4) | 1 (2) | 1 (2) | 2 (4) | |
|  |  |  |  |  | |
| Score (/15) | 6.5 (1.4) | 6.9 (1.6) | 7.6 (2.0) | 5.9 (1.1) | |
| EQ-Visual Analog Scale (/100) | 70.2 (18.1) | 65.7 (19.8) | 63.7 (18.4) | 78.5 (12.3) | |
